# Psychotropic medication use among patients with celiac disease

**DOI:** 10.1186/s12888-018-1668-0

**Published:** 2018-03-27

**Authors:** Haley M. Zylberberg, Jonas F. Ludvigsson, Peter H. R. Green, Benjamin Lebwohl

**Affiliations:** 1 Division of Digestive and Liver Diseases, Department of Medicine, Columbia University College of Physicians and Surgeons, New York, USA; 2 Department of Medical Epidemiology and Biostatistics, Karolinska University Hospital and Karolinska Institute, Stockholm, Sweden; 3 Department of Pediatrics, Örebro University Hospital, Örebro, Sweden; 4 Department of Epidemiology, Mailman School of Public Health, Columbia University, New York, USA; 5 The Celiac Disease Center at Columbia University, 180 Fort Washington Avenue, Suite 936, New York, NY 10032 USA

**Keywords:** Celiac disease, Psychiatric disorders, Epidemiology, Depression, Anxiety

## Abstract

**Background:**

Celiac disease is a multi-system disorder with manifestations that may result in psychiatric disorders. We assessed the prevalence of medication use to treat psychiatric disorders in celiac disease patients.

**Methods:**

We conducted a cross-sectional study of patients undergoing esophagogastroduodenoscopy over 9-years at a celiac disease referral center. We compared the prevalence of psychotropic medication use among celiac disease patients (*n* = 1293) to a control group (*n* = 1401) with abdominal pain or reflux.

**Results:**

Among all patients the mean age was 48.4 years, most were female (69.5%), and 22.7% used any psychotropic medication. There was no difference between overall psychotropic medication use among celiac disease patients and controls (23.9% vs 21.8%, OR 1.16; 95% CI 0.96–1.39, *p* = 0.12). However, those with celiac disease were more likely to use antidepressants on univariate (16.4% vs 13.4%, *p* = 0.03) and multivariate analysis (OR 1.28; 95% CI 1.03–1.59; *p* = 0.03). Use of psychotropic medications was not associated with disease duration or mode of presentation of celiac disease.

**Conclusions:**

Celiac disease patients use psychotropic medications at similar rates as those with other gastrointestinal diseases, though subgroup analysis suggests they may use more antidepressants. Future studies should investigate whether celiac disease is associated with mood disorders that are not treated with medications.

## Background

Celiac Disease affects up to 1% of the general population worldwide [1]. In addition to gastrointestinal symptoms and sequelae of malabsorption, celiac disease is characterized by extraintestinal manifestations including neurological and psychiatric disorders [2, 3]. There is uncertainty regarding the nature of the relationship between psychiatric illnesses and celiac disease. The reported prevalence of depression in celiac disease has varied widely, ranging from 6% to 57% [4–12], with some studies showing a positive association between celiac disease and depression [4–6, 8, 9, 11, 13–19], others showing no association [7, 10, 20–22], and still others showing no difference compared to other chronic diseases [9, 10, 18, 21]. Anxiety has been associated with celiac disease in some studies [4, 5, 13, 14], while others found no association [7, 10, 18, 23]. Even fewer studies have addressed the association between celiac disease and attention deficit hyperactivity disorder [10, 24], bipolar disorder [10, 17, 19], and fatigue [11, 25, 26].

Many of these studies have been limited by small sample sizes [4–6, 8, 9, 11, 13–16, 19, 20, 22–24, 26–29], with only a few [7, 10, 12, 17, 21, 25] including more than 200 celiac disease subjects. Furthermore, only one of these studies was performed in the United States [10]. In order to assess the prevalence of psychiatric disorders we assessed the prevalence of medication use to treat psychiatric disorders among patients with celiac disease undergoing endoscopy in a North American setting.

## Methods

In this cross-sectional study, we used an electronic endoscopy database to search all patients aged at least 18 years undergoing outpatient esophagogastroduodenoscopy (EGD) during a 9-year period from January 1st 2007 to Dec 31st 2015 performed at Columbia University Medical Center, a hospital-based endoscopy suite in New York City. The presence of celiac disease was defined based on endoscopy reports whose listed EGD indication was either evaluation of suspected celiac disease (with duodenal biopsy showing villous atrophy) or follow-up evaluation of celiac disease. We then identified a control population consisting of individuals undergoing EGD for the indications of gastroesophageal reflux disease (GERD) or for abdominal pain (including dyspepsia). To minimize underlying sociodemographic differences between the celiac disease patients and controls, we limited our inclusion criteria to endoscopies performed by the six gastroenterologists affiliated with the Celiac Disease Center at Columbia University, a major referral center for the diagnosis and management of celiac disease. As is routine practice before endoscopic procedures, all patients underwent a pre-procedure interview with a nurse where the patient was directly asked to list all current outpatient medications. The medications gathered in these interviews were the only medications used in our analysis.

For our primary exposure of interest, we compared the prevalence of any psychotropic medication use in celiac disease patients to controls. We then examined individual classes of psychotropic medications, including antidepressants, anti-psychotics, anxiolytics, mood stabilizers, sleep aids, and sympathomimetics (see appendix). As a secondary outcome, we divided patients with celiac disease according to disease duration and mode of presentation (classical symptoms of malabsorption compared to non-classical or asymptomatic cases, as previously defined in the literature) [30] so as to determine whether duration of treatment after diagnosis, as well as symptoms at the onset of diagnosis, were associated with psychotropic medication use. We calculated disease duration when date of celiac disease diagnosis was available among patient records in our prospectively-maintained celiac disease patient database.

We used the chi square and Fisher exact tests to compare proportions and calculate *p*-values and we used multivariate logistic regression, adjusting for age and sex, to calculate odds ratios (ORs) and their 95% Confidence Intervals (CIs), reporting measures of association between psychotropic medication use and the variables of interest. We also performed multiple logistic regression, adjusting for age and sex, to quantify the association between psychotropic medication use in patients with celiac disease and duration of celiac disease as well as mode of presentation. All reported *p* values are 2-sided. We used SAS version 9.4 (Cary, NC) for all analyses. Columbia University Medical Center’s Institutional Review Board approved this study, IRB-AAAQ9855, on August 9th 2016, asserting that this study met all ethical guidelines. As this study included data that had already been collected, informed consent was not deemed necessary.

## Results

During the nine-year period of the study, 1293 patients underwent EGD for celiac disease by 6 providers at the Celiac Disease Center at Columbia University. During this time period, 811 and 590 non-celiac patients underwent endoscopy by those providers for evaluation of abdominal pain and GERD, respectively. Demographic information is shown in Table 1. The mean age of the entire sample was 48.37 years. Celiac disease patients had a lower mean age compared to controls (44.9 years vs 51.6 years, *p* < 0.0001). Females made up 70% of our population and were the dominant sex in both groups, though a larger percentage of patients with celiac disease were female (73% vs 66%, *p* = 0.0003).

**Table 1:** Characteristics of study patients

Clinical Characteristics	Total (*n* = 2694) (%)	Celiac Disease (*n* = 1293) (%)	Controls (*n* = 1401) (%)	*P* value
Age (in years)				
Mean age (± SD)	48.37 (± 17.3)	44.87 (± 17.0)	51.60 (± 17.0)	< 0.0001
18–30	534 (19.2)	238 (26.1)	196 (14.0)	< 0.0001
31–40	451 (16.7)	240 (18.6)	211 (15.1)	
41–50	457 (17.0)	217 (16.8)	240 (17.1)	
51–59	464 (17.2)	205 (15.9)	259 (18.5)	
≥ 60	788 (29.3)	293 (37.2)	495 (35.3)	
Sex				
Males	823 (30.6)	352 (27.2)	471 (33.6)	0.0003
Females	1871 (69.5)	941 (72.8)	930 (66.4)	
Procedure Indication				
Celiac Disease (cases)	1293 (48.0)			
Abdominal pain (controls)	811 (30.1)			
GERD (controls)	590 (21.9)			

A comparison of psychotropic medication use in patients with celiac disease compared to controls is shown in Table 2. Almost one-fourth of the study sample reported taking at least one psychotropic medication (*n* = 614, 23%). Among those who reported any psychotropic medication use, the most common category was antidepressant use (15%), followed by anxiolytic use (8%). Among patients with celiac disease, 23.9% used any psychotropic medication, compared to 21.8% of controls (*p* = 0.12). After controlling for age and sex, celiac disease was not significantly associated with any psychotropic medication use (OR 1.16; 95% CI 0.96–1.39; *p* = 0.12). This null relationship remained when restricted to males (OR 1.27; 95% CI 0.87–1.87, *p* = 0.22), females (OR 1.15; 95% CI 0.92–1.43, *p* = 0.23), those aged 18–50 years (OR 1.20; 95% CI 0.93–1.55, *p* = 0.16), and those aged ≥51 years (OR 1.07; 95% CI 0.82–1.40, *p* = 0.61).

**Table 2:** Psychotropic medication use in patients with Celiac Disease compared to Controls

Psychotropic Medications	Total (*n* = 2694)	Celiac Disease (*n* = 1293)	Controls (*n* = 1401)	Univariate *P* Value	Multivariate* OR (95% CI)	Multivariate* *P* Value
Any	614 (22.8)	309 (23.9)	305 (21.8)	0.12	1.16 (0.96–1.39)	0.12
Antidepressants	399 (14.8)	212 (16.4)	187 (13.4)	0.03	1.28 (1.03–1.59)	0.03
Anti-psychotics	48 (1.8)	18 (1.4)	30 (2.1)	0.14	0.65 (0.36–1.19)	0.16
Sleep Aids	84 (3.1)	41 (3.2)	43 (3.1)	0.88	1.12 (0.72–1.75)	0.60
Mood Stabilizers	47 (1.7)	20 (1.6)	27 (1.9)	0.45	0.76 (0.42–1.37)	0.35
Sympathomimetics	65 (2.4)	33 (2.6)	32 (2.3)	0.65	0.94 (0.57–1.55)	0.79
Anxiolytics	210 (7.8)	100 (7.7)	110 (7.9)	0.91	1.07 (0.80–1.43)	0.65

*Adjusted for age and sex

Antidepressant use was more common in patients with celiac disease (16.4%) than controls (13.4%) on univariate analysis (*p* = 0.03), an association that remained significant on multivariate analysis when adjusting for age and sex (OR 1.28; 95% CI 1.03–1.59; *p* = 0.03). However, after removing tricyclic antidepressants (*n* = 32) from the antidepressant category, antidepressant use was not more common in patients with celiac disease compared to controls on both univariate (CD: 193 (14.9%) vs Controls: 174 (12.4%), *p* = 0.06) and multivariate analysis (OR 1.23; 95% CI 0.98–1.54, *p* = 0.07). The null relationship between celiac disease and overall psychotropic use remained when tricyclic antidepressants were removed from the overall psychotropic medication use category (CD 295 (22.8%) vs Controls 297 (21.2%), *p* = 0.13). All other psychotropic medication categories were not statistically significant on univariate or multivariate analysis.

On multivariate analysis of factors independently associated with psychotropic medication use (Table 3), females (irrespective of celiac disease status) were more likely to use psychotropic medications (OR 1.41; 95% CI 1.15–1.73, *p* = 0.001). Patients older than 50 were also more likely to use these medications compared to younger patients (OR for 51–59: 1.36; 95% CI 1.01–1.84, *p* = 0.05; OR for ≥60: 1.37; 95% CI 1.04–1.80, *p* = 0.03).

**Table 3:** Multivariate analysis of age and sex Associated with use of any Psychotropic Medication*

Variable	OR	95% CI	*p* value
Age (in years)			
18–30	1.00	–	–
31–40	1.07	0.79–1.47	0.66
41–50	1.28	0.94–1.74	0.11
51–59	1.36	1.01–1.84	0.05
≥ 60	1.37	1.04–1.80	0.03
Sex			
Males	1.00	–	–
Females	1.41	1.15–1.73	0.001

*Adjusted for age and sex

Analysis of variables associated with psychotropic medication use among those with celiac disease is shown in Table 4. Among celiac disease patients, neither age nor sex was associated with psychotropic medication use on univariate analysis and multivariate analysis. Among patients for whom disease duration was known (*n* = 556) and for whom mode of presentation was known (*n* = 689), we tested these variables for an association with psychotropic medication use. Neither duration of celiac disease nor mode of presentation was associated with psychotropic medication use on univariate or multivariate analysis. A comparison of psychotropic medication use in celiac disease patients with a new diagnosis (prior to, or within 1 month of starting the gluten-free diet) compared to those who were previously diagnosed (greater than 1 month) also revealed no association (new diagnosis: 13 (17.1%) vs previous diagnosis: 109 (22.7%), *p* = 0.1).

**Table 4:** Univariate and multivariate analysis of factors associated with Psychotropic use among patients with Celiac Disease

Clinical Characteristics	Psychotropic use (*n* = 309)	No psychotropic use (*n* = 984)	Univariate *P* Value	Multivariate* OR	Multivariate* *P* Value
Age (in years)					
Mean age (±SD)	45.35 (± 16.2)	44.72 (± 17.3)	0.57		
18–30	68 (20.1)	270 (79.9)	0.06	1.0	–
31–40	60 (25.0)	180 (75.0)		0.71 (0.35–1.47)	0.36
41–50	54 (24.9)	163 (75.1)		1.21 (0.65–2.25)	0.55
51–59	63 (30.7)	142 (69.3)		1.19 (0.63–2.27)	0.60
≥ 60	64 (21.8)	229 (78.2)		0.77 (0.42–1.43)	0.41
Sex					
Males	74 (21.0)	278 (79.0)	0.14	1.0	–
Females	235 (25.0)	706 (75.0)		1.61 (0.99–2.61)	0.05
Duration of celiac disease (*n* = 556)					
Newly diagnosed (0–6 months)	16 (18.2)	72 (81.8)	0.14	1.0	–
6 months - 1.99 years	16 (25.8)	46 (74.2)		1.60 (0.72–3.53)	0.25
2–4.99 years	22 (16.2)	114 (83.8)		0.86 (0.42–1.76)	0.67
≥ 5 years	68 (25.2)	202 (74.8)		1.44 (0.77–2.72)	0.26
Mode of Presentation (*n* = 689)					
Classical	62 (23.5)	202 (76.5)	0.35	1.40 (0.92–2.13)	–
Non-classical/silent	87 (20.5)	338 (79.5)		1.0	0.11

*Adjusted for age and sex

In a post-hoc analysis, we examined whether antidepressant use was associated with celiac disease duration, and found that antidepressant use was more common among those with five or more years of illness compared to those with four or fewer years of illness (18.15% vs 10.84%, *p* = 0.014). However, time since diagnosis was not significant on multivariate analysis (compared to newly-diagnosed patients, 6 months - 1.99 years: OR 0.82; 95% CI 0.30–2.25; 2–4.99 years: OR 0.65; 95% CI 0.28–1.54; ≥5 years OR 1.54; 95% CI 0.75–3.15).

## Discussion

We found no significant difference in psychotropic medication use between celiac disease patients and controls. Use of these medications was common in our sample, reported by 23.9% of patients with celiac disease and 21.8% of controls (*p* = 0.12). When categories of psychotropic medications were analyzed, we found that antidepressant use was more common among those with celiac disease (*p* = 0.03). To our knowledge this is the first study to measure psychotropic medication use among patients with celiac disease, and the largest study of celiac disease patients in the United States to investigate psychiatric disorders (see Table 5).

**Table 5:** Studies of psychiatric disease in CD patients and controls, where CD sample size ≥200

First author, publication year	Country	# Patients	Outcome	Results - Depression	Results - Other
Ludvigsson JF, 2007 [17].	Sweden	13,776 CD 66,815 HC	Presence of mood disorders and association between prior major depression and CD	Increased risk of subsequent depression in CD (HR 1.8; 95% CI 1.6–2.2; *p* < 0.001) Individuals with prior depression at increased risk of a subsequent diagnosis of CD (OR 2.3; 95% CI 2.0–2.8; *p* < 0.001)	CD was not associated with subsequent BD (HR 1.1; 95% CI 0.7–1.7; *p* = 0.779. Individuals with prior BD at increased risk of a subsequent diagnosis of CD (OR 1.7; 95% CI 1.2–2.3; *p* = 0.001)
Garud S, 2009 [10].	US	600 CD 200 HC 200 IBS	Psychiatric disorders	No difference in CD vs HC (17.2% vs 16.0%, *p* = 0.79) and no difference in CD vs IBS (17.2% vs 18.5%, *p* = 0.74)	No difference in anxiety in CD vs HC (8.7% vs 4.5%, *p* = 0.08) or CD vs IBS (8.7% vs 12.0%, *p* = 0.21).
Hauser W, 2010 [21].	Germany	441 CD 441 HC 235 IBD	Anxiety and Depression	No difference in all groups (*p* = 0.3) No difference in the frequency of probable depressive disorder between the three groups (*p* = 0.1)	Anxiety levels higher CD and IBD than in HC (all *P* < 0.001). No difference in CD vs IBD, when adjusted for social class (*p* = 0.3) Frequency of probable anxiety disorder higher in CDs and IBDs than in controls (15.4% vs 14.0% vs 5.7%) (all *p* < 0.001)
Mårild K, 2014 [25].	Sweden	2933 CD 14,571 HC	Hypnotic Use		4.4% CD vs 3.3% HC used ≥2 prescribed hypnotics (OR: 1.33; 95% CI: 1.08–1.62) ≥ 2 prescribed hypnotics common both ≥1 year before (OR 1.23; 95% CI 0.88–1.71) and after diagnosis (HR 1.36; 95% CI 1.30–1.41)
Present study	US	1293 CD 1401 with dyspepsia or GERD	Use of psychotropic medications	No difference in antidepressant use (OR 1.28; 95% CI 1.03–1.59; *p* = 0.03)	No difference in anxiolytic use (OR 1.07; 95% CI 0.80–1.43; *p* = 0.65), mood stabilizer use (OR 0.76; 95% CI 0.42–1.37; *p* = 0.35), or sleep aids (OR 1.12; 95% CI 0.72–1.75; *p* = 0.60)

*BD* bipolar disorder, *CD* celiac disease, *GERD* gastroesophageal reflux disease, *GFD* gluten free diet, *GI* gastrointestinal, *HC* healthy controls, *HR* Hazard Ratio, *IBS* irritable bowel syndrome, *IBD* inflammatory bowel disease

Antidepressant use was found in 16% of celiac disease patients, falling within the previously reported wide range (6–57%) of depression prevalence in celiac disease [4–12]. There is much debate about whether celiac disease is linked to depression, with some studies reporting a positive association [4–6, 8, 9, 11, 13–19] and others finding no such association [7, 10, 20–22]. This relationship is complicated by studies showing similar rates of depression in celiac disease compared to other chronic diseases, such as diabetes [9], irritable bowel syndrome [10], and inflammatory bowel disease [13, 21]. In fact, one recent meta-analysis concluded that celiac disease patients are more likely to be depressed compared to healthy controls but are just as likely to be depressed when compared to patients with chronic conditions [18]. As our control group was composed of non-celiac patients who nevertheless had symptoms that necessitated endoscopic evaluation, our control group are not an entirely healthy group. Despite this, the rates of antidepressant use in our control group (13.4%) matched the 13% use among the general US population [31], possibly reflecting a true increased association between antidepressant use and celiac disease in our study.

Any positive association between celiac disease and depression could be the result of autoimmune alterations in neurotransmitter pathways [5, 32], malabsorption of metabolic precursors such as tryptophan, folic acid, and B-12 [28, 29, 32] or perhaps due to reduced quality of life and illness burden caused by adherence to a gluten free diet [32, 33]. In our study, celiac patients who had five or more years of illness were more likely to use antidepressants compared to those who had four or less years of illness. This suggests a potential role between illness burden, possibly due to duration of treatment with the gluten free diet, and use of antidepressants. However, this relationship did not reach significance on multivariate analysis. As we did not examine rates of antidepressant use before and after the diagnosis of celiac disease or compliance with a gluten free diet, we could not quantify if symptom burden or treatment burden played a role in these increased rates.

The literature exploring the relationship between celiac disease and bipolar disorder is scarce and conflicting. This may be due to the difficulty in diagnosing bipolar disorder given that the disease encompasses a spectrum of depressive and manic symptoms and screening questionnaires have low specificity [32, 34]. In 2007, a population based cohort study performed by Ludvigsson et al. found no increased risk of bipolar disorder among 13,776 patients with celiac disease (HR 1.1; 95% CI 0.7–1.7), though they did find an increased risk of celiac disease among patients with pre-existing bipolar disorder (OR 1.7; 95% CI 1.2–2.3) [17]. In 2009, a case control study by Garud et al. also found no difference in the prevalence of bipolar disorder in 600 patients with celiac disease (2.0%) compared to 200 healthy controls (0.5%, *p* = 0.31) [10]. However, a 2015 study by Carta et al., found a higher prevalence of bipolar disorder in patients with celiac disease compared to controls: 4.3% of 60 patients with celiac disease had bipolar disorder compared to 0.4% of 240 controls (*p* < 0.005) [19]. While Ludvigsson et al. and Garud et al. used diagnostic codes to identify patients with bipolar disorder [10, 17], Carta et al. used clinical interviews, which may be more accurate [19]. In our study, we found no difference in use of mood stabilizers, which are often prescribed to treat bipolar disorder, among our 1293 patients with celiac disease compared to controls (OR 0.76; 95% CI 0.42–1.37). Nevertheless, our study may have underestimated the true number of patients with bipolar disorder because of its reliance on psychotropic medication use. For example, patients with undiagnosed bipolar disorder may first experience depression and be treated with antidepressants and only later in the course of their illness experience the manic symptoms necessary for a diagnosis of bipolar disorder and subsequent treatment with mood stabilizers. More evidence is necessary to determine if the risk of bipolar disorder is increased among patients with celiac disease.

Our study found no association between anxiolytic use and celiac disease. The lack of association between anxiety and celiac disease has been previously reported [7, 10, 18]. Studies reporting a positive association have measured subtypes of anxiety disorders, specifically subtypes related to interacting in social situations, such as social phobia [15] and panic disorder [5, 19]. Furthermore, patients with celiac disease report state anxiety [4, 13, 15], the symptom of feeling anxious temporarily, and not trait anxiety [13, 15, 23], an enduring personality trait of anxiety [35]. As our study did not use questionnaires, we could not assess the reason for anxiolytic use and any relationship between anxiety and social situations related to food.

The lack of association between overall psychotropic medication and celiac disease could be due to the makeup of our control group, who underwent EGD for either abdominal pain or GERD. It is possible that celiac disease patients have similar rates of overall psychotropic medication use compared to patients with symptoms that warrant comprehensive medical investigations, such as our control population. This would suggest that symptoms of illness play a role in subsequent diagnosis of depression. However, we found no link between psychotropic medication use and mode of presentation of celiac disease, which demonstrates that gastrointestinal symptoms may not be driving psychiatric illness or medication use. This lack of association between gastrointestinal symptoms and psychiatric illness was reported in a study where patients with celiac disease and non-celiac gluten sensitivity were exposed to three days of gluten [36]. While both groups experienced abdominal pain, both lacked evidence of depression and anxiety [36]. A second possibility for the similar rates of psychotropic medication use in both groups, could be that individuals with psychiatric problems are more likely to seek healthcare for their complaints and are subsequently more likely to undergo diagnostic testing for other medical conditions.

Another possibility for our null findings is that patients with celiac disease might have higher rates of psychiatric conditions but are less likely to take medications. As the only treatment for celiac disease is a non-pharmacologic diet, patients may be more likely to opt for non-pharmacologic treatments for their illnesses. Patients with celiac disease use less painkillers, sleep aids, and medicines for dyspepsia and more vitamins, micronutrients, and herbal products after being diagnosed with celiac disease compared to before diagnosis [37]. Patients with celiac disease may therefore have psychiatric disorders that are not fully treated as suggested in a recent study that found an increased risk of suicide in patients with celiac disease, even after adjusting for lifetime depressive disorder, anxiety disorder, and any psychiatric disorder [38].

Our study has several limitations. Firstly, as we relied on medication use rather than a validated instrument to establish a psychiatric diagnosis, we did not identify patients who were experiencing symptoms without using medications. Similarly, as many psychotropic medications can be used for multiple psychiatric diagnoses, medication use may not completely correlate with a specific psychiatric diagnosis. Secondly, though performance in a tertiary care center contributed to our large sample size of celiac disease patients, it is possible that this may have led to selection bias. The patients who seek out a tertiary center may have different characteristics, including use of psychotropic medications, compared to those in primary care facilities. However, we believe our control group is a useful comparator as it accounted for the presence of gastrointestinal symptoms and evaluation of these symptoms by invasive procedures in our two groups. We adjusted for age and sex in our statistical analysis, but we were not able to adjust for other possible confounders such as race or socioeconomic status as this was unavailable in our endoscopy record. We did limit our sample to patients who underwent endoscopies performed by the six physicians at the celiac disease center during a 9-year time period in order to eliminate any disparity in physician access or demographic factors that may have existed between our celiac disease and control groups. Furthermore, our findings concern only diagnosed celiac disease and do not necessarily apply to undiagnosed (and untreated) celiac disease. Finally, as we lacked patients’ comorbid conditions we could not explore the relationship between psychiatric illness celiac disease, and chronic medical conditions, which have been previously reported [9, 10, 13, 18, 21].

## Conclusions

Our study found no significant association between use of psychotropic medications in patients with celiac disease compared to a control group undergoing EGD for abdominal pain or GERD. People suffering from celiac disease may be comforted by the knowledge that they are not more likely to use psychotropic medications compared to other people experiencing gastrointestinal complaints. Our finding of an increased risk of antidepressant use among celiac disease patients raises the possibility that a pathophysiologic feature of the disease or a feeling of treatment burden predisposes to depression in the celiac disease population. Future studies should investigate psychiatric conditions in celiac disease using validated measures of psychiatric illness so as to determine the prevalence of these conditions and whether they are related to the adoption of the gluten-free diet.

## Appendix

### Classification of psychotropic medications into sub-categories

The following were classified as antidepressants: amitriptyline/elavil, bupropion/wellbutrin, citalopram/celexa, duloxetine/cymbalta, desipramine, desvenlafaxine/pristiq doxepin, escitalopram/lexapro, fluoxetine/prozac, fluvoxamine, mirtazapine/remeron, nortriptyline, paroxetine/paxil, milnacipran/savella, sertraline/zoloft, topramine, trazadone, and venlafaxine/effexor; as anti-psychotics: aripiprazole/abilify, droperidol, haloperidol/haldol, olanzapine/zyprexa, paliperidone/invega, prochlorperazine/compazine, risperidone/resperdal, trifluoperazine/stelazine, and questiapine/seroquel; as anti-anxiolytics: alprazolam/tafil/xanax, busiporone/buspar, clonazepam/klonopin, diazepam/valium, lorazepam, midazolam/versed, norazapam/ativan, phenobarbital, temazepam, and triazolam/halcion; as mood stabilizers: lithium/lithobid, carbamazepine/tegretol, topiramate/Topamax/topiramax, and valproic acid/Depakote/divalproex sodium; as sleep aids: dextromethorphan- doxylamine succinate/nyquil, eszapiclone/lunesta, ibuprofen-diphenhydramine/advil pm, naproxen- diphenhydramine/aleve pm, melatonin, and zolpidem/ambien/edluar; and as sympathomimetics: amphetamine/dextroamphetamine adderall, armodafinil/nuvigil, dextroamphetamine/dexedrine, dexmethlphenidate/focalin, lisdexamfetamine/vyvanse, and methylphenidate/concerta/Ritalin/daytrana patch, modafinil/provigil.

## Abbreviations

EGD: Esophagogastroduodenoscopy
GERD: gastroesophageal reflux disease
